# Stable Hydrothermal Waves at Steady State Evaporating Droplet Surface

**DOI:** 10.1038/s41598-017-16582-0

**Published:** 2017-11-24

**Authors:** Xin Zhong, Fei Duan

**Affiliations:** School of Mechanical and Aerospace Engineering, Nanyang Technological University, 50 Nanyang Avenue, Singapore, 639798 Singapore

## Abstract

Stable hydrothermal waves (HTWs) are found at a sessile ethanol droplet surface under the steady state evaporation. It is different from those which greatly decrease with the evaporation time in the transient droplet drying process. This study removes the possible effect of shrinking droplet on HTWs. An analysis of the dimensionless numbers indicates the increasingly enhanced role of thermocapillary instabilities upon raising the substrate temperature. The onset HTWs can be continuously maintained under the steady state evaporation conditions. Interestingly, the stable number of HTWs follows a linear fitting with the dimensionless factor incorporating the normalized temperature difference between the substrate and the surroundings and the droplet aspect ratio. The temperature heterogeneity of HTWs is intensified by increasing the substrate temperature. The stable HTWs exhibit the “one source-to-one sink” propagation at lower substrate temperatures. However, such directional traveling, normally presented in the transient HTWs in the drying droplet, is changed at the higher substrate temperatures due to the newly emerged sources and sinks under the steady state conditions.

## Introduction

Sessile droplet evaporation is a phenomenologically trivial but practically complicated problem^1,2^. It is influenced by multiple interrelated factors including phase change, static or dynamic wetting, Marangoni flow, buoyancy flow, etc. The developed interfacial flows can change the mass and energy transport at a droplet surface where evaporation takes place. Thermocapillary flow is driven by the surface tension gradient from a hot spot to a cold spot at the droplet surface^3^. Steady state thermocapillary flow was reported to play an important role in the energy transport at the droplet interface during the steady state evaporation^4–6^. The thermocapillary convection was also found to enhance the local evaporation flux^7^. The interfacial flow was mostly inferred from the internal convection pattern inside a droplet. Hu and Larson observed a weak thermocapillary flow in a sessile water droplet^8^, and indicated that the flow was easily eliminated by a small amount of surfactant^8,9^. From the moving of the probe inserted in a water droplet, the interfacial flow was visualized in a vacuum chamber under the steady state evaporation^10^. By employing the laser shadowgraph, the strong thermocapillary flows were displayed inside the sessile n-pentane and Freon-112 droplets^11^. The thermocapillary flow indicated by tracing particles was strong in octane in contrast to the counterpart in the water droplet^8^. Verified by the numerical calculations and experimental observations on the alcohol droplets, Ristenpart, *et al*. revealed that the orientation of thermocapillary flow was determined by the ratio of substrate thermal conductivity to liquid thermal conductivity^12^. Xu, *et al*. further pointed out that the direction of thermocapillary flow was also determined by the ratio of substrate thickness to droplet radius^13^. However, the thermocapillary flow is still difficult to be visualized directly at a liquid-vapor interface, whereas the temperature pattern variation at a droplet surface can be a key to demonstrate the flow.

Conventional hydrothermal waves (HTWs) were found in shallow liquid layers or films along which a lateral temperature difference was normally imposed^14–17^. For droplets, HTWs were often observed in the volatile liquids. The intrinsic thermocapillary instabilities at a droplet free surface were reported to be self-driven by evaporation, as Sefiane, *et al*. identified the HTW trains in the drying methanol and ethanol droplets^18,19^. HTWs were treated as bulk waves in the shrinking sessile droplets by detecting the thermal patterns at the liquid-solid and liquid-vapor interfaces simultaneously^20^. HTWs were examined in the drying ethanol droplets under the terrestrial and microgravity conditions^21,22^. Brutin, *et al*. reported the evolution of HTWs in the evaporating ethanol droplet on a heated substrate under the reduced gravity levels^21^ and stated that HTWs were self-driven regardless of the gravity levels^21,22^. The number of HTWs, used for characterizing the intensity of waves, has been probed in the sessile droplets which experienced shrinkage in evaporation. Sefiane, *et al*. demonstrated that the HTW number decreased linearly with the evaporation time of an ethanol droplet. A higher HTW number was found at a higher substrate temperature or a higher thermal conductivity of the substrate^18,23^. Sobac and Brutin demonstrated that the number of HTWs followed a power-law decay with the evaporation time under transient states^24^. Such a power-law decay was plotted under both terrestrial and microgravity conditions^22^.

To our best knowledge, most investigated sessile droplets, which exhibited HTWs at the liquid-vapor interfaces, were in transient drying processes. The droplets, freely evaporating on substrates, went through a change in shape and size until they were depleted. The instantaneous HTWs were found to attenuate with the reduced droplet volume in evaporation. Additionally, the evolving droplet shape, reflected by the continuously varying height or radius, brought uncertainties in estimating the dimensionless numbers for the onset of HTWs or the related instabilities. The effect of transient and continuous droplet size change, however, could not be excluded in examining the dependence of HTWs on the controlling parameters including substrate temperature. Whether HTWs can be maintained under steady state conditions is a still question. Motivated by the aforementioned issues, we investigate the thermal patterns at the interface of an evaporating droplet with a constant profile maintained by a continuous liquid supply at various substrate temperatures. The constant profile of the evaporating droplet excludes the impact of hydrodynamic instability caused by droplet shrinking, and hence simplifies the question to analyze the associated thermodynamic phenomena.

## Results

**Figure 1:**
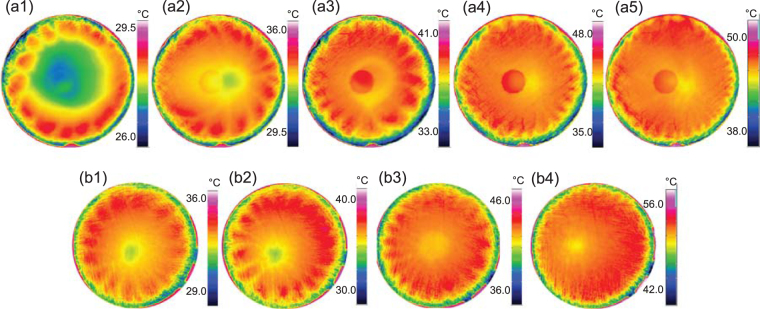
The steady hydrothermal pattern of an ethanol sessile droplet as the substrate temperature is (**a1**) 32.2 °C, (**a2**) 41.1 °C, (**a3**) 48.2 °C, (**a4**) 57.4 °C and (**a5**) 59.2 °C. Please also see Supplementary Video ES1. The instantaneous hydrothermal pattern at 15% of the lifetime of a transient ethanol sessile droplet as the substrate temperature is (**b1**) 41.0 °C, (**b2**) 46.4 °C, (**b3**) 56.2 °C and (**b4**) 64.4 °C.

Under the steady state evaporation conditions, the temperature profiles were measured at the liquid-vapor interface by an infrared camera at the different substrate temperatures from 32.2 °C to 59.2 °C, as presented in Fig. 1(a1–a5). The thermal pattern exhibits thermal oscillatory waves close to the three-phase line where the local evaporation flux is the highest. The pedal-like thermal waves distribute orthoradially with a quasi-constant angle around the axisymmetric axis of the droplet. The droplet center, at the bottom of which the fresh ethanol makes the replenishment of evaporation, is free of such thermal waves at the liquid-vapor interface, as can be seen in the infrared snapshots. As comparison, the thermal patterns captured at roughly 15% of the lifetime of the transient droplets are shown in Fig. 1(b1–b4) as the substrate temperature ranges from 41.0 °C to 64.4 °C. At 15% of the lifetime, the transient droplets have passed the initial warming up period, so the thermal pattern is relatively stable and could reflect the effect of the imposed temperature difference between the substrate and the atmosphere. It is seen that the thermal patterns are fairly alike for the steady state evaporating droplets and the transient drying droplets at the similar substrate temperatures, exemplified by Fig. 1(a2),(b1),(a3),(b2),(a4),(b3),(a5) and (b4).

The mechanism for the onset thermal patterns of the steady state droplets can be interpreted by comparing the characteristic length of the investigated problem with the thermal length expressed as ${L}_{th}={L}_{{\rm{c}}}\sqrt{\gamma /(\sigma \beta )}$ where ${L}_{{\rm{c}}}=\sqrt{(\sigma /\rho g)}$, $\gamma =d\sigma /dT$ defines the coefficient of surface tension, *β* is the thermal expansion coefficient of liquid, *σ* is the liquid-vapor surface tension, *ρ* is the liquid density, and *g* is the gravitational coefficient^25^. Stationary rolls triggered by the density difference of liquid would dominate if the characteristic length, *h* for a sessile droplet, is larger than the thermal length, *L*_th_. Otherwise HTWs are predominant. HTWs are further divided into HTW1 and HTW2 by the comparison of the characteristic length and capillary length, *L*_c_. In our study, the traveling thermal waves are categorized to HTW2 as the droplet height is always inferior to *L*_c_ = 1.69 mm. A temperature gradient is established from the three-phase line to the droplet apex as read from the infrared visualization for each experiment. A surface tension gradient therefore is developed which could generate thermocapillary instabilities. Besides, the thermogravity flow, led by the liquid density difference caused by the temperature variation from the liquid-solid interface to the liquid-vapor interface could be negligible because of the small droplet thickness.

As the droplet shape is maintained unvaried at a fixed substrate temperature, we can rely on the geometric and thermal data to estimate the relevant dimensionless numbers for characterizing the mechanism of thermal patterns. The static Bond number, $Bo=\frac{\rho g{h}^{2}}{\sigma }$, is one order of magnitude smaller than unity, suggesting that it is valid to assume a spherical cap of the droplet shape. The buoyancy and thermocapillary instabilities are respectively represented by the Rayleigh number, $Ra=\frac{\beta g{\rm{\Delta }}T{h}^{4}}{\nu \alpha R}$, and the Marangoni number, $Ma=\frac{-\frac{d\sigma }{dT}{\rm{\Delta }}T{h}^{2}}{\mu \alpha R}$, where ${\rm{\Delta }}T=({T}_{{\rm{s}}}-{T}_{{\rm{apex}}})$ is the temperature difference between the substrate and the droplet apex, *v* is the liquid kinematic viscosity, *α* is the liquid thermal diffusivity, *μ* is the liquid dynamic viscosity, and *R* is the droplet radius. As the substrate temperature, *T*_s_ = 32.2 °C, *Ra* = 61.8 and *Ma* = 1528.8, the ratio of *Ra* and *Ma* as the definition of the dynamic Bond number, $Bd=\frac{Ra}{Ma}$, is two orders of magnitude smaller than unity. It is suggested that the thermocapillary flow, much stronger than the thermogravity flow, dominates the onset of HTWs for the evaporating droplet.

**Figure 2:**
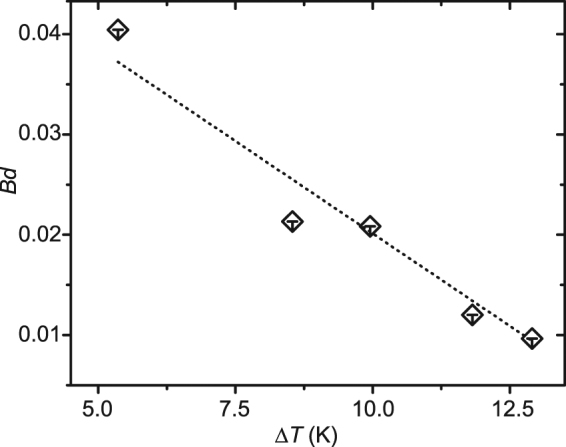
The dynamic Bond number, *Bd*, as a function of the temperature difference, Δ*T* for the steady state droplets.

Upon increasing *T*_s_, Δ*T* is larger and the droplet becomes thinner. The liquid-vapor surface tension, *σ*, is negatively dependent on temperature. The contact angle therefore is smaller at a higher substrate temperature, decreasing from 32.64° to 21.14° as *T*_s_ increases from 32.2 °C to 59.2 °C. As a result, the dimensionless numbers *Ma*, *Ra* and *Bd* are anticipated to change with *T*_s_. *Ra* and *Ma* are at an order of magnitude of 10^1^ and 10^3^ respectively at the various substrate temperatures. The dynamic Bond number *Bd*, plotted in Fig. 2, decreases conspicuously from roughly 0.04 to 0.01 as Δ*T* increases from 5.4 °C to 12.9 °C. The substantial reduction in *Bd* demonstrates the increasingly dominant role of thermocapillary instabilities inside the droplet, which is consistent with the greatly enhanced number and dynamics of HTWs at a higher substrate temperature. Interestingly, it is found that the HTW patterns can be maintained during evaporation under the steady state conditions.

**Figure 3:**
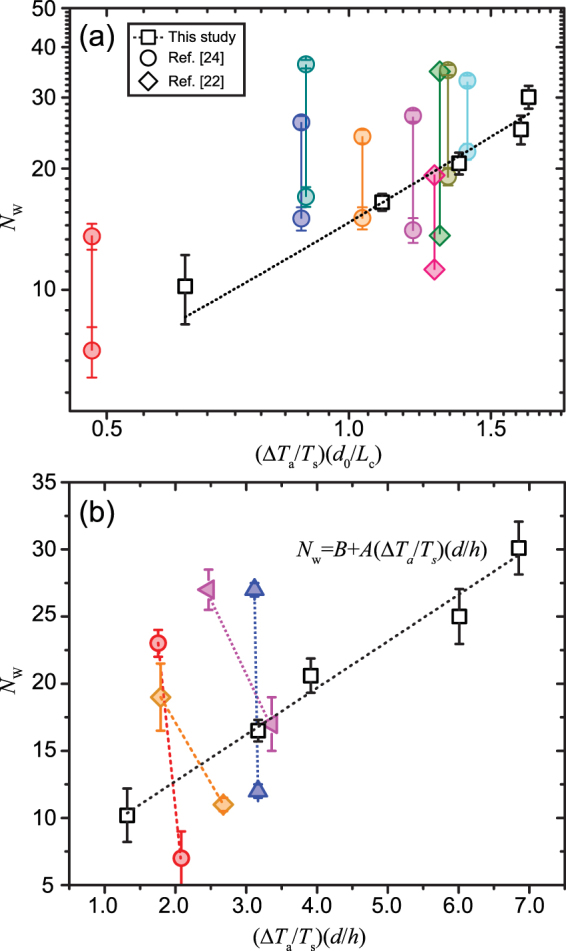
(**a**) The number of HTWs, *N*_w_, in our study for the steady state conditions, and in refs^22,24^ for the initial and ultimate moments of the droplet evaporation as a function of the log-scaled dimensionless factor, $(\frac{{\rm{\Delta }}{T}_{{\rm{a}}}}{{T}_{{\rm{s}}}})(\frac{{d}_{0}}{{L}_{{\rm{c}}}})$. The different colors of the circular symbols represent the droplets with different initial diameters and substrate temperatures in ref.^24^. The green and the rose-red rhombic symbols represent the droplets under the terrestrial and reduced gravity levels respectively in ref.^22^. (**b**) The number of steady HTWs, *N*_w_, for the steady state conditions and for the initial and ultimate moments of evaporation for the transient droplets in this work as a function of the dimensionless factor, $(\frac{{\rm{\Delta }}{T}_{{\rm{a}}}}{{T}_{{\rm{s}}}})(\frac{d}{h})$.

The repeatable thermal patterns are sampled in Fig. 1 under the steady state conditions. The stable HTWs are found to highly depend on the imposed substrate temperature such that the pattern exhibits a larger number of HTWs as *T*_s_ increases. The corresponding size of each individual HTW is smaller at a higher *T*_s_. Aimed to compare the number of HTWs, *N*_w_, with that of the transient HTWs of refs^22,24^, we employ the same dimensionless factor $({\rm{\Delta }}{T}_{{\rm{a}}}/{T}_{{\rm{s}}})({d}_{{\rm{0}}}/{L}_{{\rm{c}}})$ ^24^, where ${\rm{\Delta }}{T}_{{\rm{a}}}=({T}_{{\rm{s}}}-{T}_{{\rm{a}}})$ and *d*_0_ as the initial droplet diameter, to describe *N*_w_ at the different heating conditions. The dimensionless factor incorporates the thermal effect from substrate heating as well as the geometric effect from droplet initial wetting area. The stable *N*_w_ in the current study and the instantaneous ones at the beginning and late moments of the freely evaporating droplets with shrinking volumes in refs^22,24^ are plotted in Fig. 3(a). It can be seen that *N*_w_ of the current study has a small error bar and basically obeys a linear upward trend upon raising the dimensionless factor, revealing the enhancement in the stable *N*_w_ by increasing the substrate temperature and/or the wetting area. On the other hand, for refs^22,24^, *N*_w_ is shown by its values at the initial and late moments of evaporation, and the two values are connected by a vertical line with its length indicating the large range of the evolving *N*_w_ throughout the droplet lifetime. The transient *N*_w_ exhibit relatively high uncertainties in the shrinking droplets which could be a result of the time-dependent hydrodynamic instability due to the droplet shrinking in evaporation. However, in general, *N*_w_ in ref.^24^ basically follows an upward trend versus the dimensionless factor. Besides, it is notable that *N*_w_ in ref.^22^ is larger under the normal gravity level than that under the reduced one, but both of which basically agree with the increasing trend of *N*_w_.

Upon raising the substrate temperature from 32.2 °C to 59.2 °C, it is notable that the central height of the steady state droplet, *h*, is significantly reduced from 0.78 mm to 0.39 mm owing to the reduced *γ*_LV_. The varying *h* could have an impact on the thermal pattern and the resultant *N*_w_. To incorporate *h* in our study, the dimensionless factor is modified to $({\rm{\Delta }}{T}_{{\rm{a}}}/{T}_{{\rm{s}}})(d/h)$, of which *d* is the instantaneous droplet diameter. Herein *d* and *h* are constants for the steady state droplets, while they are time-dependent for the transient droplets in this work. The varying *h* and *d* of the transient droplets in refs^22,24^ are not taken into account in plotting Fig. 3(a) since they are not provided for the different heating conditions. $(d/h)$ fully describes the droplet geometry as $Bo < 1$ when the droplet is spherical-shaped. The variation of *N*_w_ with the new dimensionless factor is plotted in Fig. 3(b). The stable *N*_w_ exhibits a linear upward trend and it is fitted by,

1 $${N}_{w}=B+A(\frac{{\rm{\Delta }}{T}_{{\rm{a}}}}{{T}_{{\rm{s}}}})(\frac{d}{h})$$

where $A=3.47\pm 0.32$ and $B=5.79\pm 1.28$. The fitting quality of Eq. (1) is greatly improved compared to the one in Fig. 3(a), revealing the influence of droplet height on HTWs and thus the necessity of including *h* in the description of *N*_w_. The fitting reflects the dependence of *N*_w_ on the synergetic effects of the thermal factor $({\rm{\Delta }}{T}_{{\rm{a}}}/{T}_{{\rm{s}}})$ and the droplet aspect ratio $(d/h)$ under a steady state. For the transient droplets in this study, the values of *N*_w_ at the initial and late moments of evaporation are shown in Fig. 3(b) with a dashed connecting line. Resembling to the counterparts of refs^22,24^, *N*_w_ jumps greatly with the proceeding of evaporation. Due to the faster decrease in *h* than *d* of the droplets as time elapses, the trend of *N*_w_ from the initial to the late moment is rightward deviated. But in general the transient *N*_w_ basically increases with $({\rm{\Delta }}{T}_{{\rm{a}}}/{T}_{{\rm{s}}})(d/h)$ at similar moments of evaporation, e.g., the initial or the late stage of the droplet lifetime.

**Figure 4:**
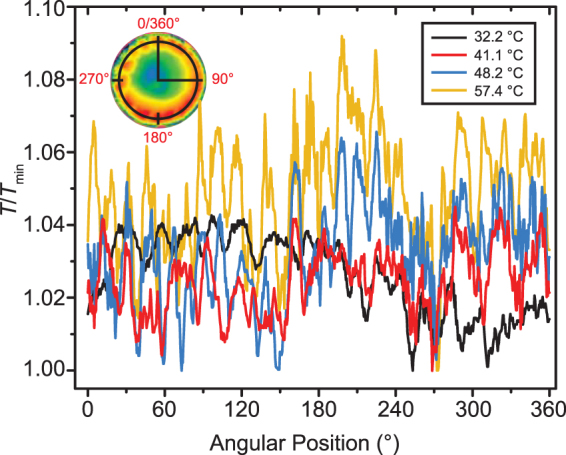
The temperature distribution at the circle depicted in the inset normalized to its minimum value, $T/{T}_{{\rm{\min }}}$, as a function of the angular position for the steady state droplets as the substrate temperature is 32.2 °C, 41.1 °C, 48.2 °C or 57.4 °C respectively.

The spatial and temperature distributions of HTWs are sensitive to the substrate temperature as well. Figure 4 shows the circular temperature depicted in the inset image of Fig. 4 normalized to its minimum value, *T/T*_min_, versus the angular position at a random time for the four steady state experiments. The circle being 0.5 mm from the perimeter is chosen to cross most HTWs for the different *T*_s_. *T/T*_min_ exhibits repeatable peaks and valleys which represent the centers and boundaries of the waves respectively. The temperature along the circle has a higher frequency of alternating peaks and valleys at a higher *T*_s_, in consistence with a greater amount of cells. Furthermore, the temperature difference between a wave center and its edge is enlarged at a higher *T*_s_. The difference of *T/T*_min_ between the wave center and the boundary is about 0.02 at 32.2 °C while it increases to roughly 0.06 at 57.4 °C. Therefore, the temperature heterogeneity of HTWs is pronounced by raising the substrate temperature.

**Figure 5:**
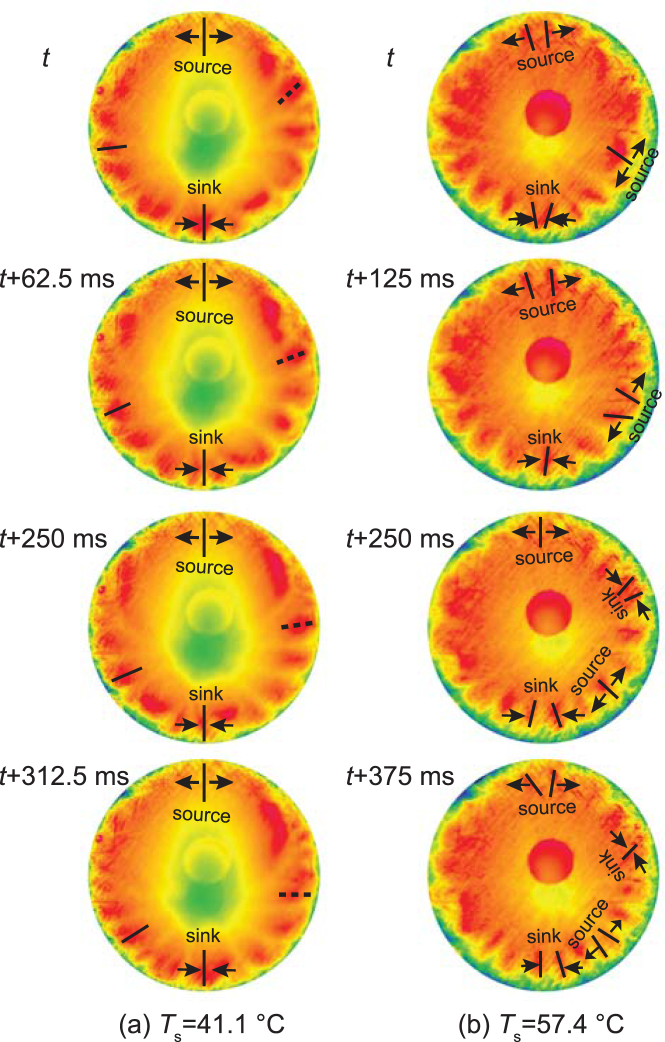
The dynamic propagation of HTWs of the steady state droplet with the substrate temperature, *T*_s_, at (**a**) 41.1 °C and (**b**) 57.4 °C.

The stable HTWs travel orthoradially in the vicinity of the three-phase line during evaporation, and their dynamics are found to be altered by the substrate temperature. We track the evolution of HTWs over hundreds of milliseconds, and focus on the sources where HTWs are generated and sinks where HTWs collapse. At a low value of *T*_s_, as exemplified by the infrared snapshots at 41.1 °C, HTWs originate from one source and join at one sink of the droplet (see Fig. 5(a) and Supplementary Video ES1). Two sets of HTWs as the examples are highlighted by the solid and dashed lines in Fig. 5(a). They propagate in either clockwise or anticlockwise direction to the sink. As *T*_s_ increases to 48.2 °C, there appears more than one sink or source. The additional sources or sinks are temporal and thus difficult to follow. The numbers of the sources or sinks are further grown as *T*_s_ is raised to 57.4 °C (see Fig. 5(b) and Supplementary Video ES1). The mono-propagation from the source to the sink is suppressed due to the occasionally emerged sources and sinks elsewhere. A new source at time, *t*, splits into the two sets of HTWs which propagate toward the left and right respectively at *t* + 125 ms. Besides, two individual HTWs at *t* + 250 ms merge together at the new sink at *t* + 375 ms. Such enhancement in the random propagation also emerges as *T*_s_ reaches 59.2 °C, but it is not shown here since small bubbles start to form and interrupt the HTWs propagation. Therefore, it is suggested that the propagation paradigm varies from “one source-to-one sink” to “multi-directional traveling” upon increasing the substrate temperature under the steady state conditions.

## Discussion

The influence of the feeding liquid on the HTW patterns can be evaluated by comparing the flow velocity in the tube connected with the upper substrate center and the typical velocity in an evaporating pure ethanol droplet. The flow velocity ranges from 0.16 mm/s to 1.67 mm/s corresponding to the flow rates as the substrate temperature is heated from 32.2 °C to 59.2 °C, whereas the mean velocity in a drying pure ethanol droplet was 8.7 mm/s under an atmospheric condition without heating^26^. Therefore, the effect of the inlet flow on the HTW pattern can be neglected at the various heating conditions. The similar HTW patterns in the steady state droplets and the transient ones in our study and in ref.^24^ suggest a little effect of the liquid supply on the stable HTWs as well. The steady thermal patterns presented in Fig. 1(a1) and (a5) with the substrate temperatures at 32.2 °C and 59.2 °C bear a great resemblance to the transient counterparts as the substrate temperature is at 34.5 °C and 60.0 °C respectively in ref.^24^. It is worth noticing that the “one source-to-one sink” propagation of the steady HTWs at the low substrate temperatures in our study resembles the HTW traveling at the various substrate temperatures in ref.^24^: HTWs originate from one source and collapse at one sink. It is also the dominant HTW traveling manner in the transient droplets of this work. It is indicated that the “one source-to-one sink” propagation is self-driven rather than being directed by the liquid supply. The lack of new sources and sinks at high substrate temperatures in ref.^24^ could be attributed to the hydrodynamic instabilities in the procedure of evaporation and the associated weakened thermal instabilities. By keeping the steady state evaporation, increasing the substrate temperature leads to newly generated sources and sinks. Therefore, the evolution from “one source-to-one sink” to “multi-directional traveling” of HTW propagation upon raising the substrate temperature should be intrinsically temperature-dependent, since we can maintain the stable temperature variation between evaporation-cooling liquid-vapor interface and the heating droplet periphery contacting with the substrate in the steady state evaporation conditions. The interesting observation shows that the onset HTWs can be maintained under steady state evaporation conditions, suggesting that the thermal pattern should be a reflection of the continuous and steady thermocapillary flow at an interface.

## Conclusion

We experimentally investigated the thermal patterns at an evaporating sessile ethanol droplet surface under steady state conditions by varying the substrate temperature. The analysis of the relevant dimensionless numbers was applied to interpret the onset of HTWs. The comparison of the HTW numbers for the steady state, the transient states in this work, and the transient states in the other two previous studies indicated that the stable HTW patterns were maintained by the steady state conditions but the transient HTWs decrease in number with the evaporation time. Under the steady state, the HTW number with a small uncertainty showed a linear increase with the dimensionless factor incorporating the normalized temperature difference between the substrate and the surroundings and the droplet aspect ratio. The temperature difference of each HTW was enlarged at a higher substrate temperature. HTWs underwent the “one source-to-one sink” propagation at lower substrate temperatures, and such directional traveling was attenuated at higher substrate temperatures due to the emergence of new sources and sinks. Increasing the substrate temperature induced more sinks and sources in the droplets. This investigation focuses on stable HTW patterns at the evaporation interface under steady state conditions, while the continuous HTWs during the whole process infer that the patterns should be a result of continuous interfacial flows in the experiments.

## Methods

**Figure 6:**
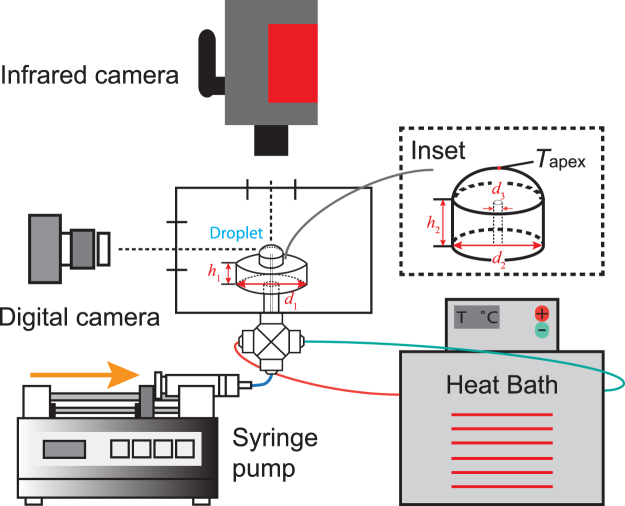
The schematics of the experimental setup. The diameter and height of the base are denoted as *d*_1_ and *h*_1_. The inset shows the schematics of the substrate above the base with the height, *h*_2_, and the diameter, *d*_2_, and the diameter of the inner tube, *d*_3_. The temperatures at the substrate perimeter, *T*_s_, and the droplet apex, *T*_apex_, are detected by the infrared camera.

The experimental system is illustrated in Fig. 6 for the evaporation of a droplet on a heated cylindrical substrate. The infrared camera (InfraTech8000, Field of View: 640 × 512 pixels, Resolution: 15 *μ*m, Measurement Accuracy: ±1 K) is fixed right above the droplet to capture the temperature profile at the evaporation interface with a rate of 80 frames per second (fps). Meanwhile, the digital camera (Canon EOS 600-D) is used to monitor the side view of the droplet at a rate of 1 fps. A test cell is adopted to enclose the evaporating droplet to avoid any disturbance of air flow. In the experiments, the geometric profiles including the contact angle, *θ*, the base diameter, *d*, and the droplet height, *h*, were post-processed by the software, ImageJ^27^. The temperature, *T*_a_, and the relative humidity, *H*_a_, of the enclosure were recorded with the fixed values at 25 ± 1 °C and 55 ± 2.5%, respectively.

As shown in Fig. 6, the cylindrical stage as a whole, made of copper, consists of two parts with the upper one as the substrate and the lower one as the base in which hot water is circulated through a heat bath (F25-EH, JULABO) to control the substrate at the predetermined temperatures. The bottom base has a diameter *d*_1_ = 15 mm and the height *h*_1_ = 8 mm. The upper substrate has a diameter *d*_2_ = 4.8 mm and height *h*_2_ = 3 mm. The upper substrate is installed with a thin copper tube with a diameter *d*_3_ = 0.5 mm across the heating stage centerline to link a syringe mounted on a syringe pump (KDS-Scientific). A sessile droplet is formed by pumping the liquid on the top of the upper substrate. The much smaller area of the substrate in conjunction with the large base is to ensure a relatively uniform distribution of the surface temperature. During the experiments, the temperature at the substrate periphery was measured by the infrared camera and used as the substrate temperature, *T*_s_. The substrate temperature was corrected by the emissivity ${\varepsilon }_{{\rm{c}}}=0.5\pm 0.02$ which was obtained from the calibration by the infrared camera for the copper surface. *T*_s_ was set with a series values higher than the room temperature but lower than the saturation temperature of ethanol, listed in Table 1. The deviation of the temperature along the substrate perimeter ranges from 0.3 °C to 1.4 °C with *T*_s_ increasing from 32.2 °C to 59.5 °C. The temperature difference between the substrate and the droplet apex is expressed as ${\rm{\Delta }}T=({T}_{{\rm{s}}}-{T}_{{\rm{apex}}})$ with *T*_apex_ as the temperature at the droplet apex measured by the infrared camera. Δ*T* is used for calculation of the dimensionless numbers at the maximum height of sessile droplets. In the calculation, we assume that the temperature at the liquid-solid interface is the same as *T*_s_ for each experiment.

**Table 1:** The flow rate, $\dot{V}$, substrate temperature, *T*_s_, the temperature difference between the substrate and the droplet apex, Δ*T*, and contact angle *θ*, at the different steady state experimental conditions.

$\dot{V}$ (mm^3^/s)	*T*_s_ (°C)	Δ*T* (°C)	*θ*(°)
0.031	32.2	5.4	32.64
0.078	41.1	8.5	32.12
0.125	48.2	9.9	30.11
0.236	57.4	11.8	27.69
0.328	59.2	12.9	21.14

Pure ethanol (Sigma-Aldrich, ACS reagent, purity >99.5%) was selected as the working fluid. Under the steady state, the injection flow rate was controlled with a constant value; the droplet configuration from the side view was maintained constant with the constant contact angle, base diameter and height; and the substrate was kept at a constant temperature. Since the surface tension of ethanol is negatively dependent on temperature, the droplet exhibited lower contact angles at higher substrate temperatures. The contact angle, *θ*, substrate temperature, *T*_s_, and temperature difference, Δ*T*, at each flow rate, $\dot{V}$, were measured in the steady state experiments, and are listed in Table 1. The droplet had a wetting diameter, *d*, at approximately 4.60 mm and the central thickness, *h*, not greater than 0.78 mm. Thus the droplet height was inferior to the capillary length, *L*_c_ = 1.69 mm. As comparison, ethanol droplets with the initial volume at 5.0 μL and the initial wetting diameter *d*_0_ at approximately 4.5 mm were evaporating onto a planar copper substrate which was heated to maintain at 41.0 °C, 46.4 °C, 56.2 °C and 64.4 °C respectively. The evolution of the droplet side profile, characterized by the central height *h* and the wetting diameter *d*, was recorded by the digital camera. Meanwhile, HTWs in the transient droplets were captured at the various surface temperatures. The HTWs in the stable and the transient droplets are compared in this article.

**Figure 7:**
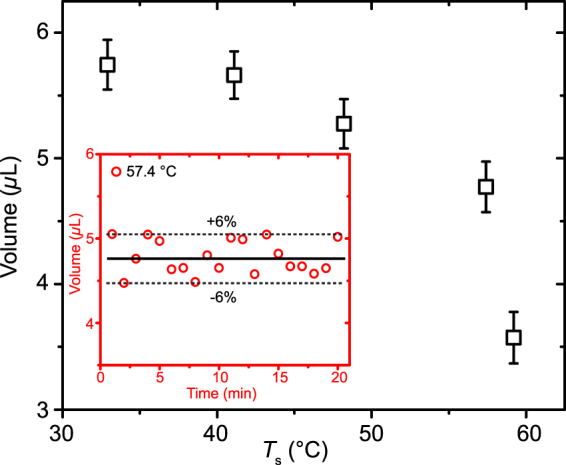
Droplet volume as a function of the substrate temperature, *T*_s_ for the steady state droplets. The inset as an example shows the variation of the droplet volume in 20 minutes as *T*_s_ = 57.4 °C.

Figure 7 presents the averaged droplet volume and its standard deviation obtained from 20 minutes of the steady state evaporation as a function of the substrate temperature. The inset of Fig. 7 as one example shows that the maximum variation of the droplet volume is within ± 6% in 20 minutes for the measurement as *T*_s_ = 57.4 °C. Generally, as seen in Fig. 7, the droplet volume decreases as the substrate temperature increases under the steady state conditions. In addition, the ethanol is semi-transparent within the infrared wavelength from 3.7 *μ*m to 4.8 *μ*m of the infrared camera. Such semi-transparency makes the infrared camera measure the average temperature over a depth adjacent to the interface within the working fluid. The temperature detected by the infrared camera in the following section is corrected by the emissivity following the formula, ${\varepsilon }_{{\rm{e}}}=1-{e}^{-a\tilde{h}}$ where *a* = 1.85 mm^−1^ and $\tilde{h}$ is the liquid thickness^24^.

## Electronic supplementary material

Supplementary Video
